# Long-Term Management of Brain Metastases From Small-Cell Lung Cancer: A Case Report of a Survivor Treated With Multiple Stereotactic Radiosurgeries

**DOI:** 10.7759/cureus.99267

**Published:** 2025-12-15

**Authors:** Michael T Milano, Deborah A Mulford, Dheerendra Prasad, Neil Almeida, Kenneth Usuki

**Affiliations:** 1 Radiation Oncology, University of Rochester, Rochester, USA; 2 Medicine, University of Rochester, Rochester, USA; 3 Neurosurgery and Radiation Medicine, Roswell Park Comprehensive Cancer Center, Buffalo, USA; 4 Radiation Medicine, Roswell Park Comprehensive Cancer Center, Buffalo, USA

**Keywords:** brain metastases, brain metastasis velocity, case report, small-cell lung carcinoma, stereotactic radiosurgery

## Abstract

There is a paucity of studies specifically addressing the treatment of brain metastases with many courses of stereotactic radiosurgery (SRS) over a long duration (i.e., years) of time. Given the morbidity of whole-brain radiotherapy (WBRT), describing the outcomes of patients treated in this manner is important. We report a case of a 48-year-old man who developed a single brain metastasis eight months after completing concurrent chemoradiotherapy for T2aN2M0, Stage IIIA small-cell lung cancer. Over the next ~13 months, each new brain magnetic resonance imaging (MRI) scan (at less than three-month intervals) revealed two to four new, asymptomatic brain metastases. Thereafter, new lesions appeared every two to three MRI scans for 17 months, after which no new lesions developed. Ultimately, he underwent nine separate SRS courses, treating a total of 21 brain metastases, over the course of two and a half years. He never developed extracranial disease progression and no brain metastases locally recurred after SRS. He developed no symptomatic adverse effects from any SRS treatment. He now remains free of disease, with his last SRS more than one year from his last follow-up imaging. This case highlights that in some patients, brain metastases can be considered a potentially chronic condition manageable with multiple courses of SRS over time, in an effort to delay or prevent the need for WBRT.

## Introduction

Over the past decade, management of brain metastases has shifted from routine whole-brain radiotherapy (WBRT) to stereotactic radiosurgery (SRS) alone, driven by the neurocognitive and quality-of-life declines associated with WBRT and the absence of an overall survival (OS) benefit [1]. Notably, however, the landmark randomized trials that established this paradigm excluded patients with small-cell lung cancer (SCLC) [1], a disease with a high propensity for central nervous system (CNS) involvement. More recent studies have evaluated the role of upfront SRS alone for brain metastases from SCLC. A 2022 meta-analysis of 31 studies reported comparable survival outcomes between SRS alone and WBRT [2]. Although SCLC typically demonstrates more aggressive behavior than non-SCLC, a large retrospective analysis found no significant differences in neurological mortality or number of progressive CNS lesions after SRS alone for SCLC vs non-SCLC. However, OS was significantly shorter in the SCLC (n=892) versus the non-SCLC (n=4,758) cohort [3].

The FIRE-SCLC study was a multicenter, international, retrospective study of 710 patients with brain metastases from SCLC treated with SRS alone [4]. Median OS were 11.0, 8.7, 8.0 and 5.5 months for patients presenting with one, two to four, five to 10 and ≥11 brain metastases, respectively. The number of metastases was significantly associated with time to CNS progression. A multi-institutional, phase II trial enrolled patients with one to 10 brain metastases from SCLC (n=96) or other small-cell cancers (n=4) treated with one- or five-fraction SRS [5]. Median OS was 10.2 months and the one-year neurologic death rate was 11.0%.

Comparing patient-level data after SRS alone (from FIRE-SCLC) versus after WBRT (from another published cohort [6]), and controlling for potentially prognostic variables, WBRT was associated with significantly improved time to CNS progression, without improvements in OS or CNS progression-free survival [4]. The NRG CC009 randomized controlled trial of SRS vs hippocampal-avoidant WBRT (HA-WBRT) for patients with SCLC brain metastases is currently recruiting.

Salvage therapy is more frequently needed in patients (not specifically with SCLC) treated for brain metastases with SRS alone as opposed to WBRT, though neurocognition and quality of life are better preserved without WBRT [1]. Thus, SRS is frequently considered for salvage, even among patients with SCLC for whom salvage WBRT remains an accepted standard of care. In the FIRE-SCLC study, 33.5% underwent subsequent SRS for recurrent/new brain metastases, whereas only 16.1% underwent salvage WBRT. In the aforementioned multi-institutional, phase II trial, 39.0% received at least one salvage SRS course and 22.0% received salvage WBRT. Neither study reported how many patients underwent multiple salvage SRS, or how many received WBRT in addition to salvage SRS. Salvage SRS may be more appropriate for those patients with low (versus high) brain metastasis velocity (BMV), which quantifies the number of new lesions that develop over time [7]. However, the use of BMV in decision-making remains investigational.

In light of the scarce literature addressing salvage management after SRS for limited brain metastases from SCLC, we aimed to characterize the outcomes of a patient who received multiple courses of SRS for brain metastases occurring across a prolonged interval. The patient has avoided WBRT and did not develop symptomatic brain metastases or adverse effects attributable to SRS.

## Case presentation

We present a patient with brain metastases from SCLC who, over the course of 2.5 years, underwent nine separate SRS courses for brain metastases detected on serial magnetic resonance imaging (MRI).

**Figure 1:**
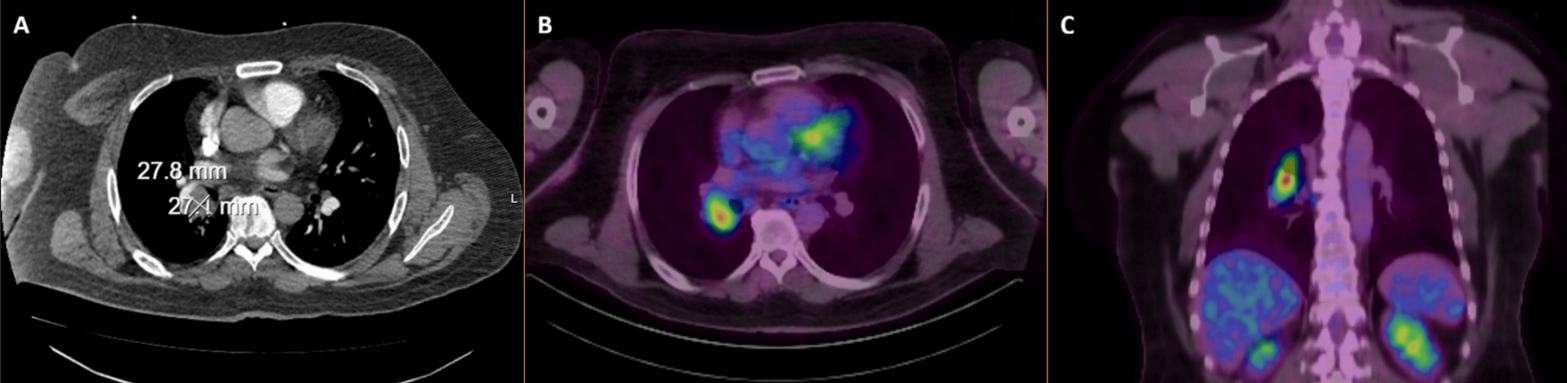
Axial image from contrast enhanced computerized tomography scan of the chest, showing a ~2 cm right hilar/lung mass (A). Axial (B) and coronal (C) fluorodeoxyglucose positron emission tomography (PET) images showing PET avidity of this lesion.

At 48 years, he was diagnosed with T2aN2M0, Stage IIIA SCLC after presenting with cough and hemoptysis. He had smoked three-quarters of a pack daily for 32 years and quit two years before SCLC diagnosis. Past medical history of gastroesophageal reflux disease and low back pain was non-contributory; he was diagnosed (in retrospect) with chronic obstructive pulmonary disease. Computerized tomography (CT) and fluorodeoxyglucose (FDG) positron emission tomography (PET)/CT revealed a hypermetabolic 2.8 x 2.7 cm right lung and hilar mass (Figure 1), with no other regional nodal or distant metastatic sites.

**Figure 2:**
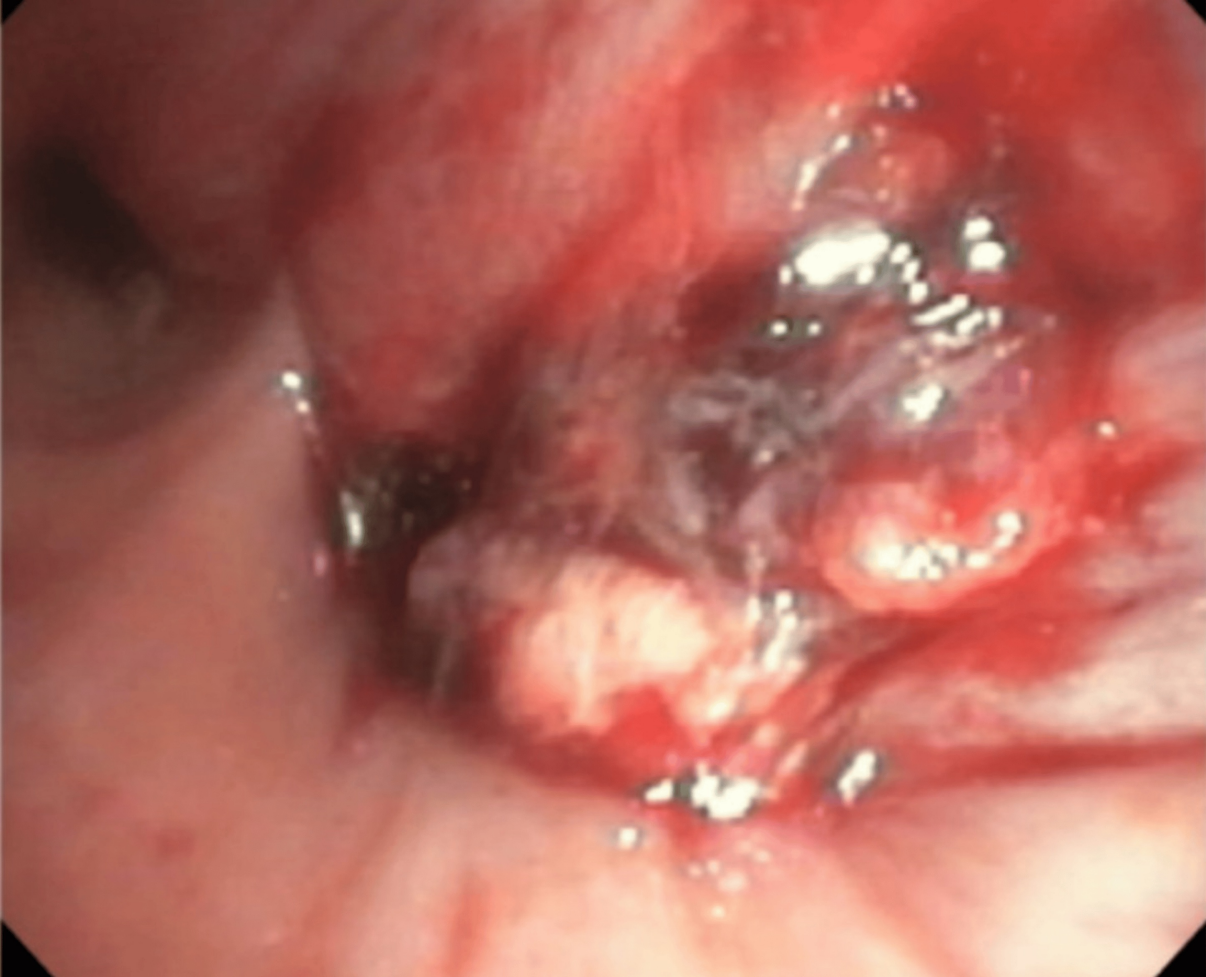
Endobronchial lesion seen at the takeoff of the right lower lung bronchus

Bronchoscopy identified an endobronchial lesion located proximal to the right lower lobe bronchus (Figure 2). The lesion was debulked, and ultrasound-guided biopsies were obtained from thoracic 4R and 7 lymph node stations. Pathology revealed SCLC from the right perihilar mass and level 4R lymph node; Ki67 was >90%. Brain MRI showed no evidence of metastases.

At diagnosis, his Karnofsky performance scale was 90 and he was working full-time in construction. The patient enrolled in the NRG LU005 clinical trial [8] and underwent concurrent radiotherapy and chemo-immunotherapy (cisplatin, etoposide, and atezolizumab). Radiotherapy and chemotherapy started on the same day (i.e., radiotherapy started with cycle 1 of chemotherapy). He received 66 Gy in 33 daily fractions, planned and delivered with volume-modulated arc therapy. Due to neutropenia, etoposide was dose-reduced for the last of four cycles. After completing chemoradiotherapy, we discussed standard and hippocampal-avoidant prophylactic cranial irradiation (PCI) ± memantine and surveillance MRI without cranial radiotherapy. He opted for the latter. He received only one cycle adjuvant atezolizumab due to development of pneumonitis attributed to immunotherapy and radiotherapy.

A chest CT scan, two weeks after completing chemoradiotherapy, demonstrated radiographic response of the SCLC and a new, non-occlusive pulmonary embolus (which has been managed with anticoagulation). Thereafter, chest, abdomen and pelvis CT scans were performed every ~three months for more than three years, before body imaging was spaced out less frequently. PET/CT scans were performed ~11 and 21 months following chemoradiotherapy to further evaluate radiographic changes in the lung. Low PET avidity was most consistent with post-treatment fibrotic lung changes. To date, there is no evidence of locoregional recurrence or extracranial metastases. Thus, salvage chemotherapy was not recommended for him.

**Figure 3:**
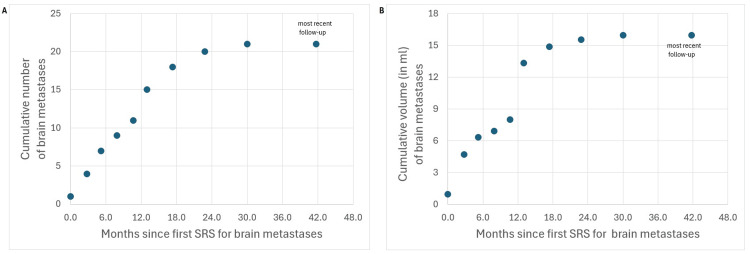
Cumulative number (A) and volume (B) of brain metastases developing over time, from the date of first stereotactic radiosurgery (SRS), in a patient with small cell lung cancer. The nine datapoints from 0 to 30 months are at the time of an SRS procedure (~two weeks from planning MRI scan), while the last datapoint at 42 months represents the most recent follow-up (at which time the patient had no new brain metastases and was clinically stable).

Pre- and post-treatment brain MRI scans were performed with a 3 Tesla scanner and included post-contrast BrainLAB® (BrainLAB, Munich, Germany) spoiled gradient recall and CUBE® (GE Healthcare, Chicago, IL, USA) sequences with 1 mm slice thickness. He developed a single ~1.2 cm right frontal brain metastasis 10.5 months from pathologic diagnosis of SCLC, eight months from completing chemoradiotherapy, three months from a brain MRI that showed no intracranial disease, and two months from receiving atezolizumab. He underwent a three-fraction SRS course. Over the next ~13 months, each new brain MRI scan (at less than three-month intervals) revealed two to four new, asymptomatic brain metastases. Thereafter, new lesions appeared every two to three MRI scans for 17 months, after which no new lesions have developed. No treated brain metastasis locally recurred. Before each SRS course, HA-WBRT was presented as an option, but he chose to proceed with SRS. To date, he has had nine SRS procedures treating a total of 21 brain metastases with a cumulative gross target volume (GTV) of 16 ml. Table 1 and Figure 3 summarize his treatments as well as the cumulative number and volume of brain metastases over time. The BMV gradually decreased to zero (no new lesions over 12+ months).

**Table 1:** Summary of nine stereotactic radiosurgery (SRS) courses for a patient with brain metastases from small cell lung cancer GTV = gross target volume; BMV = brain metastasis volume measured in number of new lesions per year; V20=volume of brain (plus target) receiving 20 Gy or more; PTV = planning target volumes Some lesions exhibited slight growth on MRI scans nine months (‡), 11 months (*), 12 months (†), 15 months (¥) after SRS, after which they eventually regressed in size on serial MR imaging

SRS	Months since first SRS	Months since last SRS	Number of lesions	BMV (#/year)	Prescribed Dose	Lesion 1	Lesion 2	Lesion 3	Lesion 4	Net GTV	Brain V20 for course
						(GTV)	(GTV)	(GTV)	(GTV)		
						[PTV]	[PTV]	[PTV]	[PTV]		
1st	-	-	1	-	9 Gy x 3	left frontal *	-	-	-	0.95 ml	2.53 ml
						(0.95 ml)					
						[1.97 ml]					
2nd	2.8	2.8	3	13.1	9 Gy x 3	right temporal †	right parietal ¥	left parietal	-	3.77 ml	12.78 ml
						(3.58 ml)	(0.13 ml)	(0.06 ml)			
						[6.14 ml]	[0.48 ml]	[0.29 ml]			
3rd	5.2	2.4	3	15	9 Gy x 3	right frontal ‡	left frontal	left temporal	-	1.64 ml	4.28 ml
						(1.41 ml)	(0.13 ml)	(0.09 ml)			
						[2.15 ml]	[0.32 ml]	[0.25 ml]			
4th	7.9	2.7	2	8.8	6 Gy x 5	left cerebellum	right frontal	-	-	0.58 ml	3.62 ml
						(0.41 ml)	(0.17 ml)				
						[1.09 ml]	[0.61 ml]				
5th	10.7	2.8	2	8.7	6 Gy x 5	right frontal	right frontal	-	-	1.08 ml	3.41 ml
						(0.70 ml)	(0.38 ml)				
						[1.20 ml]	[0.72 ml]				
6th	13	2.3	4	20.6	6 Gy x 5	right cerebellum	right temporal	left frontal †	right parietal	5.34 ml	19.46 ml
						(4.16 ml)	(0.82 ml)	(0.34 ml)	(0.03 ml)		
						[6.73 ml]	[1.75 ml]	[1.40 ml	[0.80 ml]		
7th	17.4	4.4	3	8.2	6 Gy x 5	right parietal	right frontal	left occipital	-	1.54 ml	5.55 ml
						(1.15 ml)	(0.28 ml)	(0.11 ml)			
						[1.76 ml]	[1.31 ml]	[0.92 ml]			
8th	22.8	5.5	2	4.4	6 Gy x 5	right frontal	right temporal	-	-	0.65 ml	3.75 ml
						(0.57 ml)	(0.08 ml)				
						[1.35 ml]	[0.36 ml]				
9th	30	7.2	1	1.1	6 Gy x 5	right occipital	-	-	-	0.43 ml	1.77 ml
						(0.43 ml)					
						[0.81 ml]					

**Figure 4:**
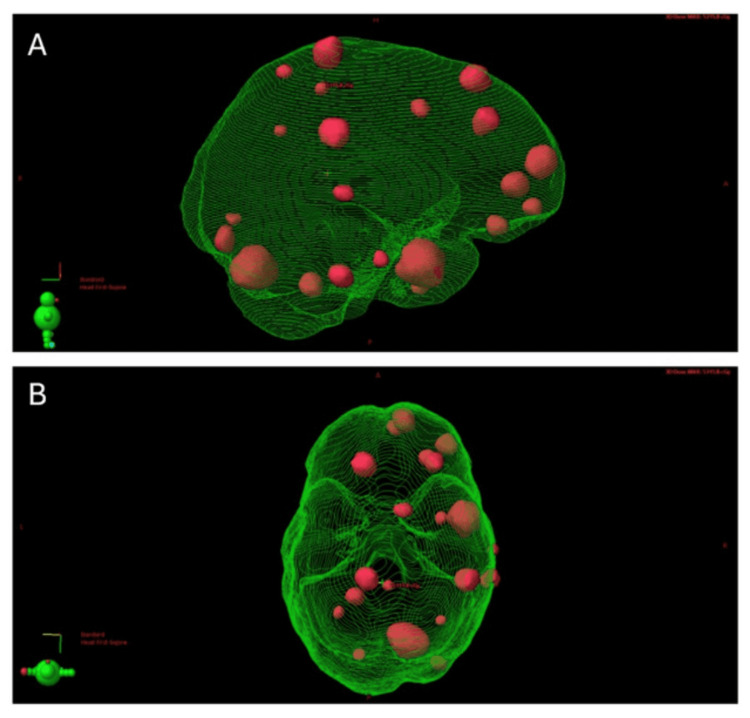
Composite planning target volumes (red) depicted in three-dimensional rendering of brain (green) in lateral (A) and vertex (panel B) views. The location of the composite maximum dose (5315.8 cGy) is shown.

A composite plan, including all nine courses of SRS, was generated to review cumulative doses to organs at risk (Figure 4). For whole brain (including targets), the composite (over nine SRS courses) mean dose, and volumes receiving >20 and >25 Gy (V20 and V25) were 10.5 Gy, 121.3 ml and 71.1 ml, respectively. Composite maximum doses to the optic apparatus (chiasm and nerves), brainstem and hippocampi were 12.1, 9.1 Gy - 8.3 Gy, respectively; minimum doses to the hottest 0.035 cc (D0.035) were 10.6 Gy, 8.4 Gy and 7.3 Gy, respectively.

Our SRS treatment approach for brain metastases is described in a prior publication [9] and briefly summarized here. Cranial 4Pi® (BrainLAB) immobilization masks were used for immobilization and the Multiple-Metastases Elements (MME)® (BrainLAB) platform was used for planning. GTVs were expanded 1-2 mm to create planning target volumes (PTV). Treatment plans were generated with dynamic conformal arcs, with >95% of each PTV receiving prescribed dose. Treatment was delivered on a Varian Edge® (Palo Alto, CA, USA) linear accelerator equipped with oblique orthogonal X-ray imagers and a six-degree-of-freedom robotic couch.

Notably, our patient never developed symptomatic brain metastases or any post-SRS toxicity. Some intervening MRI scans showed slight, asymptomatic growth of some lesions (as described in Table 1 footnotes) without appreciable MR perfusion (grade 1 necrosis). These lesions subsequently regressed on serial imaging. He was never prescribed corticosteroids for central nervous system symptoms, though he had been (up until after his last SRS) on prednisone (doses of 20-100 mg daily) to manage post-treatment pneumonitis. At last follow-up, 42 months from his first SRS and 12 months from his last, he reported no neurologic or cognitive deficits, though he has never had formal baseline or post-treatment cognitive testing. He continues to work full time (construction, landscaping and developing small businesses) with his work capacity and activities of daily living impacted by multifactorial pulmonary decline, not currently requiring supplemental oxygen.

## Discussion

While SRS alone is a standard of care for newly diagnosed SCLC brain metastases [10], studies specific to salvage therapies after SRS are lacking. SRS seemed to be the most viable approach for our patient, who never developed more than four new brain metastases. The alternatives were resuming immunotherapy, which posed significant risks to his pulmonary function and is investigational as a sole modality for SCLC brain metastases, or WBRT, which would likely detriment his quality of life and neurocognition, possibly with severe risks [11]. While the ongoing NRG BN009 study is investigating SRS vs hippocampal-avoidant whole brain radiotherapy for patients (including those with SCLC) with high BMV after prior SRS, those who previously had a BMV of four or more lesions/year are not eligible. In other words, individuals with high BMV and treated with SRS alone for newly developed brain metastases following prior SRS are excluded if new lesions subsequently appear.

It is unclear why our patient developed new lesions every few months for more than two and a half years. Perhaps the ‘new’ lesions were present early in the disease course and occult. Or possibly occult extracranial disease continued to seed new brain metastases. Either situation would be unexpected for SCLC, which tends to exhibit aggressive growth.

The BMV for our patient was initially >13 lesions/year, which is generally considered high [7] albeit typical for SCLC [12]. The trajectory of his disease progression has now slowed to the point of no extracranial disease (in >3.5 years) or intracranial progression (in more than one year). This is remarkable having had no systemic therapy or immunotherapy since prior to the initial diagnosis of brain metastases. While continued immunotherapy may have reduced our patient’s BMV, a retrospective analysis showed that immunotherapy did not decrease the risk of new brain metastases from SCLC [13].

The number of metastases treated (per course and total) with SRS in our patient was not exceptional [14]. Yet, the number of SRS courses prior to stabilization of disease is noteworthy, particularly for SCLC which is considered neurotropic and often managed with PCI and WBRT. In hindsight, one might consider early WBRT to have been nihilistic given our patient’s ultimate decelerating BMV. However, the concept of brain metastasis acceleration/deceleration [7] to refine decision-making requires further study, particularly for SCLC. One could question the cost-effectiveness of many SRS courses, but the potential debilitating effects of WBRT would weigh heavily in quality-of-life adjusted measures. Cumulative brain exposures from multiple SRS sessions is another concern, though Rivers et al. found lower whole brain exposures after multiple SRS vs after WBRT [15]. One could also argue that SRS alone may have specifically been well-suited to our patient with low-volume brain metastases that were fortuitously asymptomatic, and that, for other patients, salvage WBRT might prevent symptomatic intracranial progression.

## Conclusions

There is a paucity of studies specifically addressing patients treated with many courses of SRS for new brain metastases. Given the morbidity of whole-brain radiotherapy, describing the outcomes of patients treated in this manner is important. We highlighted a treatment approach of SRS alone for multiple, distant-recurrent brain metastases from SCLC. We described how the brain metastasis velocity accelerated and then plateaued in our patient. Ultimately, he underwent nine separate SRS courses over the course of two and a half years, and now remains free of disease. Patients, physicians, and insurers should recognize that brain metastases can be a chronic condition for some individuals. For selected patients, repeated courses of SRS over time may represent a more appropriate management strategy than WBRT. At least one patient is immensely thankful for that approach.
